# Location of capture sufficiently characterises lifetime growth trajectories in a highly mobile fish

**DOI:** 10.1186/s40462-025-00541-w

**Published:** 2025-03-17

**Authors:** Joshua S. Barrow, Jian D. L. Yen, John D. Koehn, Brenton Zampatti, Ben Fanson, Jason D. Thiem, Zeb Tonkin, Wayne M. Koster, Gavin L. Butler, Arron Strawbridge, Steven G. Brooks, Ryan Woods, John R. Morrongiello

**Affiliations:** 1 Biosciences 4, The University of Melbourne, Parkville, VIC Australia; 2 Department of Energy, Environment and Climate Action, Arthur Rylah Institute for Environmental Research, 123 Brown Street, Heidelberg, VIC Australia; 3 Gulbali Institute, Charles Sturt University, PO Box 789, Albury, NSW Australia; 4 Commonwealth Scientific and Industrial Research Organisation (CSIRO), Glen Osmond, SA Australia; 5 New South Wales Department of Primary Industries, Narrandera, NSW Australia; 6 South Australian Research and Development Institute, West Beach, SA Australia; 7 Queensland Department of Agriculture and Fisheries, Brisbane, QLD Australia; 8 Queensland Department of Environment and Science, Brisbane, QLD Australia

**Keywords:** Freshwater, Murray–Darling Basin, Otolith microchemistry, Partial migration, River regulation

## Abstract

Variation in somatic growth plays a critical role in determining an individual’s body size and the expression of its life history. Understanding the environmental drivers of growth variation in mobile organisms such as fishes can be challenging because an individual’s growth expression integrates processes operating at different spatial and temporal scales. Traditionally, otolith (ear stone) based growth analyses have focussed on temporal environmental variation by assuming an individual spends its whole life at its capture location. This approach ignores the movement potential of individuals and thus the role of spatio-temporal variation in conditions experienced. Here, we develop a modelling framework that incorporates individual movement information reconstructed via the analysis of chemical tracers in otoliths. We assess whether consideration of movement histories is important to estimating growth of a mobile freshwater fish, golden perch (*Macquaria ambigua*) at three spatial resolutions: *basin-scale*, *reach-scale (movement-exclusive)*, and *reach-scale (movement-inclusive)*. The predictive capacity of annual growth models slightly improved from the basin to the reach spatial scales (inclusive or exclusive of movement histories). Contrary to expectations, incorporating individual movement information, did not improve our ability to describe growth patterns. Golden perch growth was linked to the magnitude of and variation in spring, summer, and previous-year (antecedent) discharge, and spring temperature. The direction and magnitude of these effects was, however, dependent on life stage. Adults benefitted strongly from any increase in discharge or temperature, whereas juveniles benefitted only from increased summer discharge and grew slower in years characterised by wetter and warmer springs. We suggest that, for highly mobile fish like golden perch and in the absence of fine, ‘within reach’ scale biological data, coarser ‘reach-scale’ environmental variation may adequately describe individual growth trajectories.

**Supplementary Information:**

The online version contains supplementary material available at 10.1186/s40462-025-00541-w.

## Introduction

An individual’s growth rate is sensitive to the environmental conditions it experiences across its life [8, 43]. This environmental experience naturally varies through time and can be further modulated by movement through the landscape. Although movement is energetically expensive [11], it provides animals the opportunity to change the environmental conditions that they experience [4]. Traditional approaches to modelling growth generally ignore individual movement history and instead assume a fixed location throughout an individual’s life [47, 73], likely because undertaking long-term tagging projects and obtaining time-varying life history parameter estimates are expensive and practically difficult to implement [1, 13]. Such paucity of data precludes the detailed assessment of how spatio-temporal environmental variation affects growth.

**Fig. 1:**
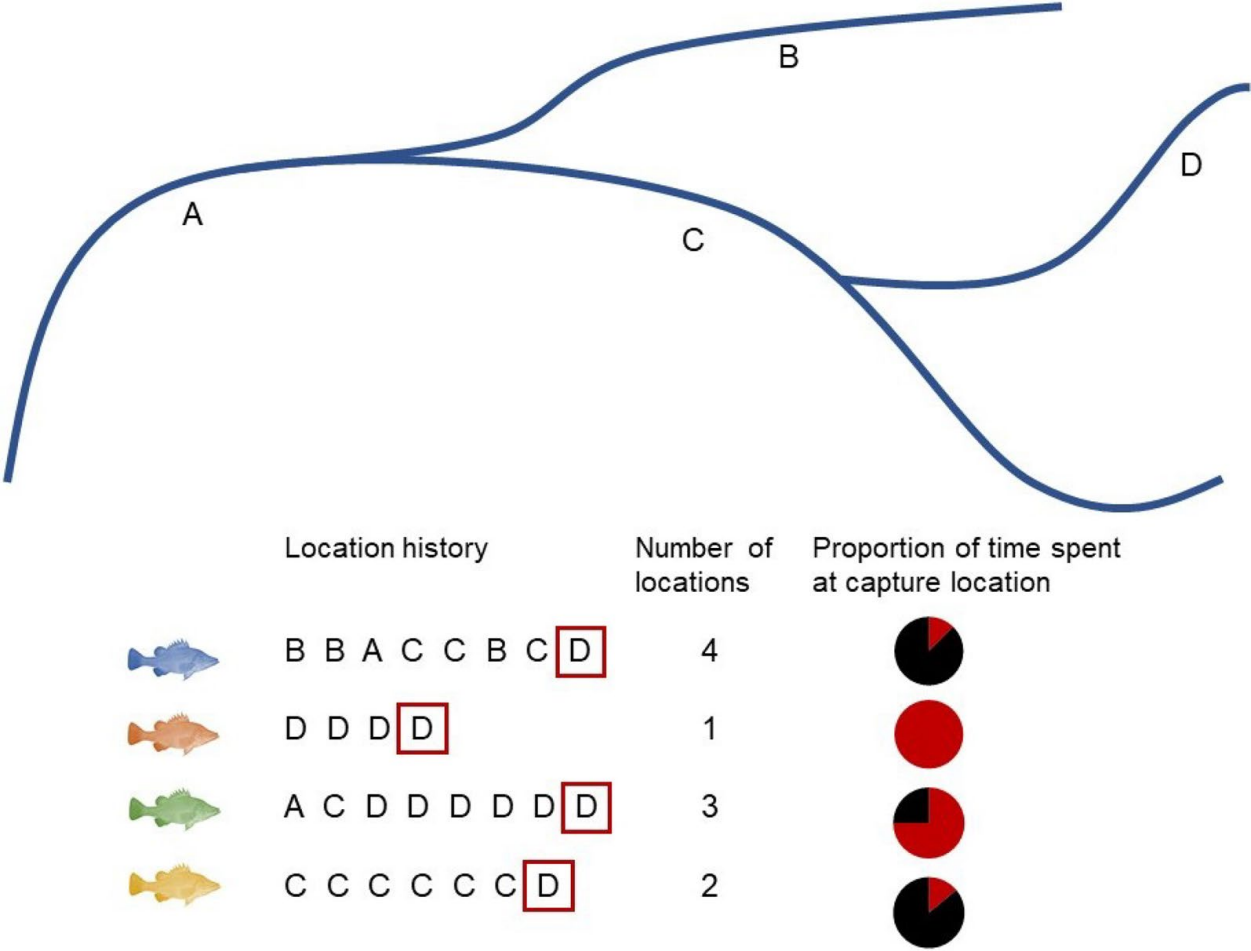
Conceptual representation of the capture location and spatio-temporally resolved location models compared in this study. The letters represent different locations within the river system. We highlight four individuals with different lifetime movement histories among the four river locations. All individuals inhabited a different number of locations, but were all captured in the same location, D, shown by the red squares. The pie charts represent the proportion of time that each individual spent at their capture location

The resolution at which environmental predictors influence somatic growth depends on the interplay between an individual’s genotype, the spatial pattern and extent of environmental variation that they experience, and how that individual moves through the environment [18]. In some cases, regionally resolved environmental drivers may explain much of the variation in individual growth, especially when environmental conditions are highly correlated across larger spatial scales [28]. This situation may occur in environments that exhibit distinct seasonal variation or those prone to episodic climatic events where regional processes outweigh small-scale differences among habitats [9, 10]. However, regional environmental drivers may not capture more localised differences in environmental conditions that are important for growth [58]. Further, individuals that move may experience many different local environmental conditions throughout their life [61]. In these cases, linking individual growth to environmental predictors may require both finely resolved environmental variables and information on an individual’s location throughout their life (Fig. 1).

For many animals, it is difficult to retrospectively identify where they have previously lived [24, 32]. Consequently, analyses attempting to identify the environmental determinants of individual growth typically rely on regional climatic conditions or environmental data from locations where individuals are assumed to have lived, such as their capture location [44, 47]. Although these methods have been used successfully for both marine and freshwater species [6, 69], they do not address the potential uncertainties that arise when individuals of wide-ranging species move long distances from their capture locations (Fig. 1). Otoliths (ear stones) in fish present a possible solution to this challenge, in that they naturally archive growth and movement information across an individual’s lifetime [17]. Combining movement and growth information in otoliths has the potential to improve fish growth models, especially in highly mobile species (e.g. [7]).

Rivers provide an excellent opportunity to test how the resolution of environmental predictors affects fish growth, as species inhabiting rivers are exposed to conditions that vary both spatially and temporally in an essentially linear environment [57, 74]. Climatic processes, such as the El Niño-Southern Oscillation in Australia, can drive large-scale anomalies in water temperature and discharge levels [21]. This regional-scale environmental variation can have wide-ranging impacts on conditions dictating growth, such as promoting system productivity and habitat accessibility in years of flooding [20]. In contrast, environmental variation can be specific to individual reaches within larger river basins, such as local fluctuations in discharge and water temperature, the presence of barriers, and prey abundance [25]. These local-scale processes can have considerable influence on fish growth by affecting physiological demands and access to feeding opportunities [27, 46, 69]. Importantly, most management interventions are focussed at the more local reach scale, so understanding the relative role of regional versus local environmental conditions has significant implications for the scale at which future management regimes are designed [26, 72].

In this study, we explored how environmental drivers operating at different spatio-temporal scales affect the growth of a highly mobile fish, golden perch (*Macquaria ambigua*) in the Murray–Darling Basin (MDB) in eastern Australia. As the prevalence of partial migration of this species creates potential discrepancies between the actual locations occupied by individuals at a given time and those inferred from their capture locations (Fig. 1), we used otolith-derived data to determine whether an individual’s growth is best explained by regional, local, or movement-inclusive local environmental conditions. Specifically, we compared environmental predictors representing three spatio-temporal resolutions (Fig. 1): *basin-scale*, encompassing regional climatic conditions affecting the entire MDB, *reach-scale (movement-exclusive)*, the current technique using environmental conditions from the capture location, and *reach-scale (movement-inclusive),* including environmental conditions from actual locations of individuals in each year of their lives. In so doing, our study addresses fundamental links between growth, movement, and environmental conditions. We also present a methodological advance in how we model individual fish growth, by testing the implicit assumption in most growth models that fixing an individual’s location to the place of capture is sufficient to characterise the environmental conditions experienced over its lifetime.

## Methods

### Study region, sample collection and preparation

Our study encompassed 10 of the 22 major catchments in the Murray–Darling Basin (MDB) in eastern Australia. The MDB covers 1,073,000 km^2^, and contains two of the longest rivers in Australia: the Murray River (2530 km in length) and the Darling River (2245 km in length) [42]. We focussed on 11 capture locations in river reaches from across the MDB that vastly differ in climatic and hydrological characteristics (Table 1): the lower Murray River, mid Murray River, upper Murray River, Condamine-Balonne Rivers, Macquarie River, Murrumbidgee River, Loddon River, Goulburn River, Campaspe River, lower Darling River, and the mid Darling River (Supp. Figure 1). Fish were collected between 2013 and 2018 and ranged from 2 to 26 years of age. The study therefore spanned a period of considerable hydrological variation, including the longest drought on record (Millenium Drought 2001–2009 [70]) followed by large, drought-breaking floods in 2010–2011.

**Table 1:** The total number, age and size range of golden perch collected from locations across the MDB, the average, minimum and maximum number of increments measured per year and location combination, and a summary of hydrological and climatic conditions

Capture Location	Number of fish	Year range of growth measurements	Growth increments per year × location (mean & range)	Age range (years)	Size range (mm)	Mean annual flow (ML/Y)	Climatic conditions From Larkin et al. [40]
Lower Murray River	146	1998–2017	42.4 (6–141)	2–21	134–515	5,052,505	Wet winter, low summer rainfall
Mid Murray River	109	1998–2017	36.4 (1–98)	2–20	240–522	3,173,092	Wet winter, low summer rainfall
Upper Murray River	87	1992–2017	27.2 (1–84)	3–25	273–549	4,332,736	Wet winter, low summer rainfall
Lower Darling River	43	2009–2017	25.8 (7–29)	2–10	95–451	629,869	Seasonally uniform rainfall
Mid Darling River	31	2008–2017	14.7 (1–31)	3–11	298–479	1,216,745	Seasonally uniform rainfall
Loddon River	12	1997–2017	4.4 (1–12)	2–22	305–518	56,240	Wet winter, low summer rainfall
Goulburn River	46	1995–2017	12.6 (1–40)	2–24	171–520	1,000,958	Wet winter, low summer rainfall
Campaspe River	20	2005–2017	5.6 (1–20)	2–14	220–490	117,905	Wet winter, low summer rainfall
Macquarie River	6	2011–2017	5.3 (5–6)	3–8	319–397	61,994	Seasonally uniform rainfall
Condamine-Balonne River	20	2009–2017	11.3 (3–20)	2–10	214–462	217,655	Wet summer, low winter rainfall
Murrumbidgee River	39	1998–2017	14.2 (1–39)	3–21	295–500	2,441,447	Wet winter, low summer rainfall

We sourced 559 golden perch otoliths from previous studies conducted by Zampatti et al. [79], Zampatti et al. [78], and Zampatti et al. [76] (Table 1). A 400–600 μm thick transverse section was prepared from each otolith for stable isotope and annual growth increment analyses, allowing us to recreate individual movement and growth histories.

### Quantifying movement and growth

We took a digital image of each otolith using a CCD digital camera mounted onto a Leica M80 dissecting microscope at 16 × magnification. We estimated the age of each individual by counting the opaque zones on the dorsal side of the otolith section [2]. We re-aged a subsample of the otoliths (n = 123) to calculate the average percent error (APE) of ageing estimates, a common measure of precision in age estimation. Next, we measured the distance between the outer edges of sequential opaque zones to estimate annual growth rates [17]. We did not include the first annual increment, as the width of this increment can vary due to factors unrelated to growth, such as spawning date and sample preparation.

Previous work [78, 79] had already analysed transects of strontium isotope ratios (^87^Sr/^86^Sr) in otolith sections using laser ablation inductively coupled mass spectrometry (LA-ICP-MS). Dissolved ^87^Sr/^86^Sr in water is primarily derived from the underlying geology of the local and upstream areas and can provide a geographically unique marker in otoliths [36]. Otoliths were ablated along a transect from the primordium to the outer edge to reveal movement histories of individuals throughout their lives.

To estimate individual locations, we used an assignment algorithm described in Zampatti et al. [76], and Zampatti et al. [77], that is based on an algorithm developed by Brennan and Schindler [12]. The algorithm used a regression tree approach, which began at the location of capture and assigned a probability that each stationary section in an otolith ^87^Sr/^86^Sr profile was from any of the possible river reaches. The algorithm divided otolith ^87^Sr/^86^Sr profiles into non-transitionary sections. Working backwards from the capture location, the algorithm assigned a probability that each non-transitionary section was formed in each river reach, by comparing the average ^87^Sr/^86^Sr from the stationary period in the otolith transect with the average ^87^Sr/^86^Sr from each river reach. The algorithm then considered the distance of each reach from the current location of the individual, by using a multiplier that decreased with distance to essentially rule out reaches that aren’t possible for a fish to have moved to and to assign a higher probability to nearby reaches. In cases where the fish had moved location within a growth increment, the location with the higher percentage of growth was selected. The algorithm also included an option for a hatchery natal origin, whereby individuals were assigned as a hatchery fish if ^87^Sr/^86^Sr matched that of the water at a hatchery and the ^87^Sr/^86^Sr profile of the fish had a sharp change between 200 and 800 μm from the core of the otolith (within approximately the first two months of life after which individuals are stocked into the wild). All assignments were reviewed and validated by experts familiar with the study species and relevant system. Approximately 92% of model-derived spatial allocations were deemed correct by experts, with the remaining 8% of individuals having at least one manual change to their annually resolved location. Changes may have occurred when, for example, the algorithm predicted a fish to be residing in a reach when actual environmental conditions such as low flow levels made instream barriers impassible and thus precluded a fish moving into the reach.

### Statistical analysis

We used a series of linear mixed effects models to relate annual growth (otolith increment width, mm) to environmental predictors measured at each of three spatial scales. Fixed effects were *age*, *life stage*, and *age class*. We included age to describe the strong effect of age on growth, and included life stage as an interaction term with the environmental predictors because we expected environmental conditions to have different effects on the growth of juveniles (2 years old), sub-adults (3–4 years old), and adults (> 4 years old) [38, 64]. Age class was the age at which an individual was captured and was included to account for any biases in growth rate associated with differential mortality or unintentional age selectivity in the capture of individuals [48].

We included a series of random effects specific to each model. At all three spatial scales, we included a random intercept and slope that allowed the effect of age to differ among individuals (age | fish identity). This term also accounted for the non-independence of increments formed in otoliths of the same fish. The basin-scale model included a random intercept for year (1 | year) to account for non-independence of increments formed by different fish in the same year, and had the following structure:

#### Basin-scale

- growth ~ (life stage × basin-scale environmental predictors) + 
- age + age class + 
- (age | fish identity) + 
- (1 | year).

The reach-scale (movement exclusive) model included a random intercept for capture location to account for differences in growth among capture locations (1 | capture location), and a nested random effect of years within capture locations to account for spatio-temporal variation in growth (1 | capture location: year):

#### Reach-scale (movement exclusive)

- growth ~ (life stage × capture location environmental predictors) + 
- age + age class + 
- (age | fish identity) + 
- (1 | capture location) + 
- (1 | capture location: year).

The reach-scale (movement inclusive) model included a random intercept for annual location to account for spatial differences in growth (1 | annual location) and a random intercept to account for differences in growth among natal locations (1 | natal origin). This model also included a nested random effect of years within annual locations to account for spatiotemporal variation in growth (1 | annual location: year):

#### Reach-scale (movement inclusive)

- growth ~ (life stage × spatio-temporally resolved environmental predictors) + 
- age + age class + 
- (age | fish identity) + 
- (1 | natal origin) + 
- (1 | annual location) + 
- (1 | annual location: year).

### Environmental predictors

We used a different set of environmental predictors at each of the three spatial scales. Predictors were defined by water years (Jul 1–Jun 30) to ensure that each variable captured the key fish growing seasons, particularly over the Austral summer (Dec–Feb) that extends across multiple calendar years and when water temperature is higher and food is more abundant. The basin-scale model included the southern oscillation index (SOI) for each year and the annual air temperature anomaly for the MDB region. SOI and temperature anomaly data were sourced from http://www.bom.gov.au/climate/. The SOI is a measure of El Niño and La Niña events in the Pacific Ocean, with negative values (El Niño) reflecting warmer and dryer conditions and positive values (La Niña) reflecting cooler and wetter conditions across eastern Australia [14]. The mean annual temperature anomaly describes the deviation of annual mean temperature from the long term (1961–1990) mean temperature [14].

The two reach-scale models used the same environmental variables but measured at different locations. The movement-exclusive model used predictor variables measured at the location of capture, while the movement-inclusive model used predictor variables measured at annually resolved locations inhabited by individuals. The reach-scale models included water temperature in spring (Sep–Nov) (spring temperature) and summer (Dec–Mar) (summer temperature), median daily discharge in spring (Sep–Nov) (spring discharge) and summer (Dec–Mar) (summer discharge), the coefficient of variation in spring discharge (Sep–Nov) (spring discharge variability) and summer discharge (Dec–Mar) (summer discharge variability), and maximum discharge in the previous (antecedent) water year (antecedent discharge). We divided median daily discharge (for the specified months) and maximum antecedent discharge by the median of maximum annual discharge at each location from 1991–2019. We standardised variables in this way to improve comparability of discharge metrics among rivers that differed substantially in size. We standardised by the median of maximum values rather than median discharge because the median discharge in the intermittently flowing Condamine-Balonne Rivers was 0 ML for some years during the reference period (1991–2019). Water temperature data were only available for some years and reaches so missing values were estimated from a modelled relationship between water temperature, date, river discharge, and air temperature data (Supp. Table 1.). All data were sourced from:

http://data.water.vic.gov.au/static.htm, https://riverdata.mdba.gov, https://water-monitoring.information.qld.gov.au/, https://realtimedata.waternsw.com.au/, and http://www.bom.gov.au/climate/data/.

We examined the collinearity between environmental predictors with pairwise Pearson’s *r* and excluded models that included two variables that had a correlation greater than 0.7 [22]. Spring and summer temperature exceeded this pairwise correlation (Supp. Figure 2) and so were included only in competing models. To support interpretation of model comparisons we used linear regressions to estimate correlations between environmental predictors from the movement-exclusive and movement-inclusive models, that is, comparing environmental predictors for each individual assuming they do not move from their capture location to those where movement was allowed. In addition, we used a correlation matrix to identify spatial correlations in environmental predictors among locations in the MDB.

### Model selection and validation

We used model selection based on Akaike’s Information Criterion (AICc) corrected for small sample size to identify the highest-ranking models at each spatial scale, where the model with the lowest value was deemed to be the most parsimonious [15]. First, we compared all models within each spatial scale, then compared the best model from each of the three spatial scales against each other. We inferred strong support for any models within 2 AICc of the top-ranked model (equivalently, ΔAICc < 2) [16]. We fitted models with maximum likelihood for initial model selection and refitted the highest-ranked combination of fixed and random effects at each spatial scale with restricted maximum likelihood (REML) to produce unbiased parameter estimates. We fitted models using the lme4 package (Bates et al*.* 2015) in the program R (v3.6.2) [59] and compared them using the AICcmodavg package [45].

We approximated the predictive capacity of the fitted models at each spatial scale using tenfold cross validation [62]. Ten-fold cross validation involves splitting the data into ten equal-sized folds, fitting the model to the data set with each fold removed, and using the fitted models to predict growth in the held-out fold. We split out data into ten groups of equal numbers of fish, which included all increments associated with each fish. We calculated cross-validated model fits using the marginal R^2^ [53], which assesses model fit at the population level (i.e., without knowledge of random effect levels in the hold-out data).

## Results

**Fig. 2:**
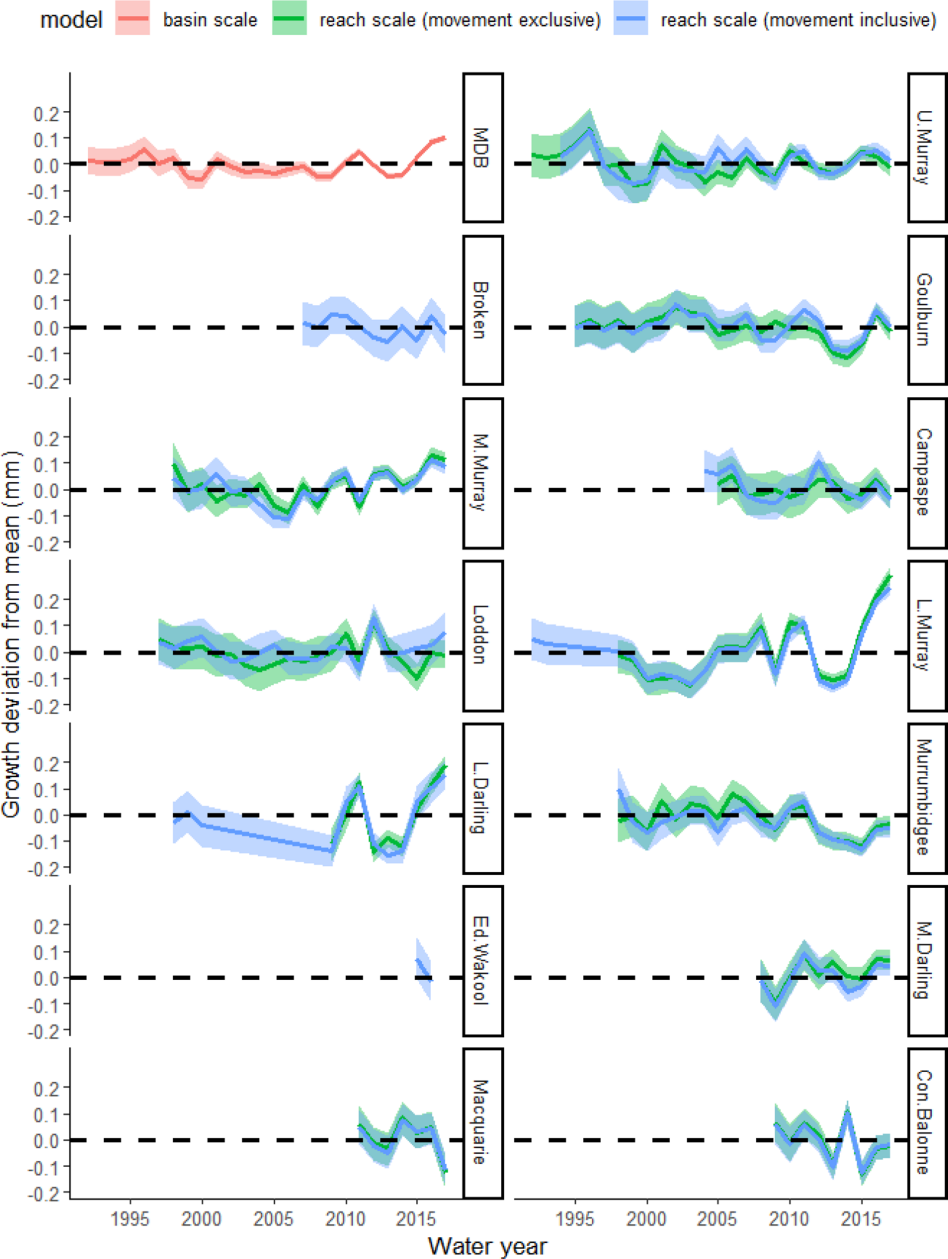
Plot of temporal growth deviations (best linear unbiased predictors [BLUPs] ± SE) among river reaches, with positive and negative values indicative of years with faster/ slower growth than average (horizontal dotted line). The red line is the average growth deviation of individuals across the Murray–Darling Basin. The green line is growth from the fixed movement-exclusive locations and the blue line is growth from the spatio-temporally variable movement-inclusive model

We measured 3538 annual otolith increments formed between 1992 and 2017 in 559 golden perch (Table 1). The average number of growth increments that were measured for each year and capture location combination and included in the models ranged between 4.4 and 42.4 (Table 1). The average percent error from the precision analysis was 1.05%, and 84.55% age estimates were agreed across readings. Based on the assignment algorithm, 51% of fish moved at least once in their life and 49% remained resident, and 82% of the annual growth increments were formed within the capture location, while 18% were formed in another location (Supp. Table 2). There were 24 otolith increments that were assigned to two river reaches (the Broken River, and the Edward-Wakool River system) that were not from any of the reaches where fish sampling occurred. Across all models, the fixed effect of age explained the most variation in growth, with growth rates (otolith growth increments) declining as age increased (Supp. Table 3). There was also an effect of age class, with individuals captured at younger ages having faster growth rates (Supp. Table 3). Growth rates differed among years but showed similar annually resolved patterns in the movement-exclusive and movement-inclusive models (Fig. 2).

The top-ranked basin-scale model included both the interaction between life stage and SOI and the interaction between life stage and temperature anomaly (Table 2). This model had a cross-validated R^2^ equal to 0.78, which was 0.01 more than the base model that excluded environmental effects (Table 2). There was a weak, negative effect of SOI for juvenile and sub-adult growth and a weak, positive effect of SOI on adult growth (Supp. Figure 3). There was a stronger negative effect of annual temperature anomaly on juvenile growth and a positive effect on adult growth (Supp. Figure 3). The second highest-ranked basin-scale model had ΔAICc of 4.

**Table 2:** Comparison of models including environmental conditions from the three different spatial scales

Environmental predictors	df	AICc	ΔAICc within spatial scale	ΔAICc across spatial scale	Cross-validated R^2^
*Basin-scale models*					
Life stage × (SOI + Temp anomaly)	**16**	**− 670.70**	**0.00**	**419.75**	**0.78**
Life stage × Temp anomaly	13	− 666.46	4.24		0.79
Life stage × SOI	13	− 646.48	24.22		0.79
Null model	8	− 615.76	54.94		0.77
*Reach-scale models (movement exclusive)*					
Life stage × (Spring discharge + Summer discharge + Antecedent discharge + Spring discharge variability + Summer discharge variability + Spring temperature)	**29**	− **1090.45**	**0.00**	**0.00**	**0.81**
Life stage × (Spring discharge + Summer discharge + Antecedent discharge + Spring discharge variability + Spring temperature)	26	− 1090.37	0.08		0.81
Life stage × (Spring discharge + Summer discharge + Antecedent discharge + Spring temperature)	23	− 1090.16	0.30		0.81
*Reach-scale models (movement inclusive)*					
Life stage × (Spring discharge + Summer discharge + Antecedent discharge + Spring temperature)	**24**	− **1053.42**	**0.00**	**37.03**	**0.81**
Life stage × (Spring discharge + Summer discharge + Antecedent discharge + Summer discharge variability + Spring temperature)	27	− 1052.01	1.40		0.80
Life stage × (Spring discharge + Summer discharge + Antecedent discharge + Summer temperature + Spring temperature)	27	− 1049.91	3.51		0.80

The left column describes the environmental conditions in all basin-scale models, and the five best movement exclusive and movement inclusive location models. The three right columns are the degrees of freedom in the model (df), the AICc value, ΔAICc value, and marginal R^2^ values based on tenfold cross validation. The top ranked model from each model comparison is shown in bold

The AICc value of the best movement-exclusive model was over 400 units less than the best basin-scale model, which revealed that including environmental variables with finer resolution improved model fit (Table 2). Four models had strong support among the movement-exclusive models that were compared (ΔAICc < 2). The top-ranked model included proportional spring and summer discharge, variability in spring and summer discharge, antecedent discharge, and spring water temperature (Table 2). This model had cross validated R^2^ equal to 0.81, which suggests that it had only slightly higher predictive capacity than the best basin-scale model (Table 2). The other three models that had strong support included different combinations of the same predictors as in the top-ranked model (Table 2).

Including individual movement histories and adding spatially resolved random effects did not appreciably improve the performance of the growth models relative to that of the movement-exclusive models (Table 2). The top-ranked, movement-inclusive model included proportional spring and summer discharge, antecedent discharge, and spring water temperature (Table 2). The other model with ΔAICc < 2 from the movement-inclusive model comparison included the same predictors as the best model with the addition of summer discharge variability (Table 2). Despite having a larger AICc value than its movement-exclusive counterpart (ΔAICc = 37), the top-ranked movement-inclusive model had an identical cross validated R^2^ value of 0.81, indicating that both models had the same predictive capacity (Table 2).

**Fig. 3:**
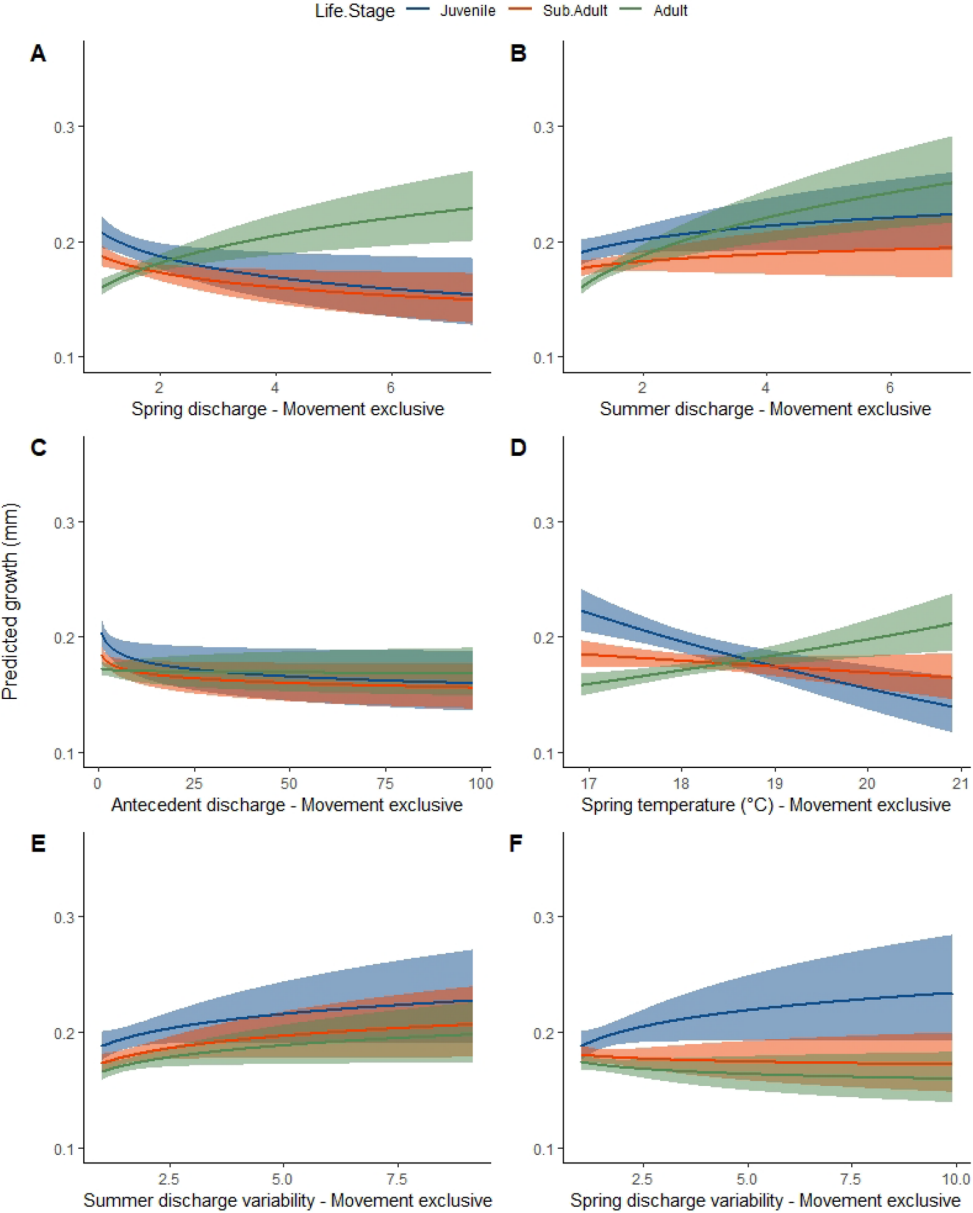
The predicted effect (± 95% CI) of relative values of **A** spring discharge, **B** summer discharge, **C** antecedent discharge, **D** spring temperature, **E** variability in summer discharge, and **F** variability in spring discharge on life stage-specific golden perch growth (otolith increment, mm), as derived from the best temporally resolved reach-scale (movement exclusive) model. The blue lines are juvenile growth, the orange lines sub-adult growth, and the green lines adult growth

Golden perch growth differed among years and among locations, with similar fitted trends and estimated environmental effects in the top-ranked movement-exclusive and movement-inclusive models (Fig. 3; Supp. Figure 4). Growth of juveniles was positively associated with summer discharge, spring and summer discharge variability, and negatively associated with spring discharge, antecedent discharge, and spring temperature (Fig. 3; Supp. Figure 4). Growth of adults was positively associated with spring discharge, summer discharge, antecedent discharge, summer discharge variability, and increased spring temperatures, but was negatively associated with spring discharge variability (Fig. 3). Growth of sub-adults was negatively associated with spring discharge, antecedent discharge, spring discharge variability, and spring temperature, and was positively associated with summer discharge and summer discharge variability (Fig. 3; Supp. Figure 4).

Environmental predictors used in the movement-exclusive and movement-inclusive models were positively correlated (Pearson’s *r* = 0.61–0.96; Supp. Figure 5). Environmental predictors were highly correlated among reaches within the southern region of the MDB, including reaches of the Murray River and tributaries, and within the Barwon-Darling River system in the north of the basin (Supp. Figure 6). There were, however, negative correlations between antecedent discharge and between summer variable discharge in the northern and southern reaches of the MDB (Supp. Figure 6).

## Discussion

Our study used information naturally archived in golden perch otoliths to identify links between individual growth and regional, inferred local, or actual local environmental conditions. Although reach-scale environmental predictors outperformed basin-scale predictors, incorporating information on individual movements over a large spatial scale did not yield further improvements in model performance. Here, we discuss our findings in relation to golden perch ecology before exploring the wider implications of movement and an individual’s environmental experience on models of fish growth.

### Environmental determinants of growth in a highly mobile, freshwater fish

Spring water temperature was positively associated with adult growth. Fast growth in response to increased water temperature has been recorded in many fish species [46, 49], likely explained by close association between water and fish body temperatures [33]. Water temperature influences several important determinants of somatic growth, such as metabolic rate, digestion rate, muscle activity, and reproductive energy allocation [3, 41]. Surprisingly, spring water temperature was negatively associated with juvenile and sub-adult growth. Although consistent with a recent study of another, less-mobile MDB freshwater fish [64], the mechanisms driving decreased juvenile growth rates in higher temperatures are unclear. These negative associations may reflect changes to metabolic activity in different temperatures, or shifts in the abundance, size, and behaviour of important macroinvertebrate prey species at warmer temperatures, which would influence food quality and availability and, consequently, growth rates [56].

Increased spring and summer discharge were positively associated with growth of adult golden perch, likely due to boosts in productivity stimulated by increased river discharge [31]. These effects were quite pronounced in our data set due to the Millennium Drought between 1997–2010 [52], followed by the significant floods of 2011 and in 2016/17 (Fig. 3). The productivity of lower order consumers and prey species of predatory fish in large rivers are supported by organic carbon originating from both instream sources and inundation of surrounding floodplains [34, 71]. The positive associations between growth and antecedent discharge reflect these productivity pathways, while also reflecting potential lags in productivity pulses from low to high trophic orders [5, 69]. The positive association with summer discharge variability indicates that positive growth outcomes can also be achieved in low flow years, possibly as a result of discharge variability promoting improvements in water quality and increases in food availability through intermittent wetting of productive habitats [65, 69].

Growth of juveniles and sub-adults had similar, positive associations with summer discharge but were negatively associated with spring discharge. Juveniles may prefer shallow, low-velocity habitats and access to these areas may be limited during high spring flows [30, 64]. Similarly, discharge above critical thresholds can curtail feeding by inhibiting individual movement at local scales [67]. The links between growth and discharge may also depend on the direction of movement and the spatial distribution of food resources, with individuals swimming against or with high discharge and encountering regions with differing abundances of prey [19, 54]. The effects of high spring discharge are more likely to be prevalent in the southern regions of the MDB, which is where the majority of our samples were collected and where higher discharge is more regularly observed. The interactions between discharge and channel geomorphology in the northern regions of the MDB is more complex due to the abundance and location of floodplain habitat and variable timing of rainfall events.

### Movement, spatial resolution, and individual growth

Knowledge of the locations an individual inhabited across its life, at least at the resolution possible for our analysis, did not improve growth model performance relative to equivalent models based on a single, inferred location. This finding suggests that in the absence of fine-scale, ‘within reach’ environmental data, golden perch growth responds to broader, reach-scale environmental conditions that are sufficiently characterised without needing information on the annual location of individuals (light-grey shaded region in Fig. 1). Environmental conditions are generally correlated across space and time [39, 50], and these correlations are likely to be exacerbated in river systems due to their directional and interconnected nature [35, 66]. In our study system, environmental conditions were correlated more strongly between river reaches that were geographically close or highly connected (Supp. Figure 6). Additionally, we observed limited movement between the geographically distinct and disconnected southern and northern reaches of the MDB. Given that wide-ranging and highly mobile animals can integrate environmental signals at multiple scales, from local habitat patches to entire regions [44, 58], we suggest that highly mobile species may have similar environmental experiences even over large spatial extents [60, 68, 75]. This may not, however, be the case for animals that move across distinct environmental gradients, such as diadromous fishes.

Processes operating at fine spatial or temporal resolutions, such as the availability of microhabitats and short-term, local movements, are likely to be highly relevant to individual growth. However, no methods currently exist to easily recreate these processes for historical, long-term datasets. Indeed, such data may never become available, particularly over the often large temporal and spatial scales relevant to mobile species. A lack of finely resolved environmental data is often suggested as a key factor leading to poor model performance or inaccurate results [23, 63]. In contrast, our study suggests that growth patterns of long-lived, highly mobile species may, in some instances, be better represented by coarser, reach-scale environmental conditions. Importantly, it is these reach-scale conditions that are most often targeted by management interventions [29]. In the case of regulated rivers, reach-specific discharge may be manipulated to target aspects of individual performance, be it growth, reproduction, recruitment, or survival [37, 51, 55], to facilitate the sustainable management of freshwater fish populations.

## Supplementary Information

Additional file 1.

## Data Availability

Data from this analysis are provided via Figshare at: https://figshare.com/s/b881d75005bddf673d50 and are strictly for reproduction purposes.
