# Supplementary material for: Location of capture sufficiently characterises lifetime growth trajectories in a highly mobile fish
Source: Mov Ecol. 2025 Mar 17;13:18. doi: 10.1186/s40462-025-00541-w (PMC11912647; doi:10.1186/s40462-025-00541-w)
Supplement: Supplementary file 1 — Additional file 1. [file 40462_2025_541_MOESM1_ESM.docx]

**Supplemental Information for:**

### Location of capture sufficiently characterises lifetime growth trajectories in a highly mobile fish

**Table of Contents:**

| **Predicted water temperature data** | Page 2 |
| --- | --- |
| **Counts of fish movement** | Page 3 |
| **Parameter estimates from most parsimonious models** | Page 4 |
| **Map of the Murray-Darling Basin** | Page 7 |
| **Correlations between environmental predictors** | Page 8 |
| **Predicted effects from the best coarse-scale model** | Page 9 |
| **Predicted effects from the best reach-scale (movement**  **Inclusive) model** | Page 10 |
| **Correlations between the same environmental**  **predictors from different movement-inclusive and**  **movement-exclusive models** | Page 11 |
| **Spatial correlations between environmental predictors** | Page 12 |

# *Predicted daily water temperature data*

Water temperature data was only available at a small number of gauges, and for limited years. Using the observed water temperature data, we developed a linear model to predict daily water temperature missing at other times and locations. Observed water temperature was the response variable, and we included the additive effects of four predictors: day of the year, month, daily river discharge (ML/day), and minimum daily air temperature. We used the log of river discharge (including the addition of a small constant to account for zero discharge days) so that discharge variability was comparable across less variable and more variable reaches. We calculated the R^2^ value for the model (Supp. table 1).

| **Model Description** | **R^2^** |
| --- | --- |
| Water temperature ~ Month + Day + Air Temp + log(River discharge+1) | 0.91 |

Supp. table. 1. Predicted water temperature model structure and associated R2 value.

Supp. table. 2. Counts of fish movement based off the ^87^Sr/^86^Sr assignment algorithm. Columns are movement categories, the total count, and the percentage of counts from each category.

| **Movement description** | **Number** | **Percentage** |
| --- | --- | --- |
| *Movement & residency* |  |  |
| Fish that moved at least once during their life | 286 fish | 51.2% |
| Fish that remained resident | 273 fish | 48.8% |
| *Otolith increment formation* |  |  |
| Total number of increments formed at capture location | 2912 increments | 82.3% |
| Total number of increments formed at a location other than capture location | 627 increments | 17.7% |

Supp. table. 3. Parameter estimates (with SE) and test statistic (*t*) from the top-ranking model from the basin scale, reach scale (movement exclusive) and reach scale (movement inclusive) model comparisons.

| Parameter | Estimate | SE | *t* |
| --- | --- | --- | --- |
| **Basin scale model** |  |  |  |
| *Fixed effects* |  |  |  |
| Intercept | -1.51539 | 0.049676 | -30.506 |
| Age | -0.67777 | 0.029608 | -22.891 |
| Age class | -0.05522 | 0.021674 | -2.548 |
| Life stage (sub adult) | -0.1427 | 0.031721 | -4.499 |
| Life stage (adult) | -0.25836 | 0.034918 | -7.399 |
| SOI | -0.00244 | 0.002986 | -0.817 |
| Temp anom | -0.14889 | 0.050561 | -2.945 |
| Life stage (sub adult) : SOI | 0.001372 | 0.002415 | 0.568 |
| Life stage (adult) : SOI | 0.0052 | 0.002268 | 2.293 |
| Life stage (subadult) : temp anom | 0.067729 | 0.035547 | 1.905 |
| Life stage (adult) : temp anom | 0.159639 | 0.03366 | 4.743 |
| *Random effects* |  |  |  |
| SD(Fish ID) | 0.111 |  |  |
| SD(Age\|Fish ID) | 0.249 |  |  |
| Cor(Age\|Fish ID) | 0.560 |  |  |
| SD(Year) | 0.055 |  |  |
| **Reach scale model (movement exclusive)** |  |  |  |
| *Fixed effects* |  |  |  |
| Intercept | -1.63531 | 0.02515 | -65.022 |
| Age | -0.71306 | 0.024858 | -28.685 |
| Age class | -0.02987 | 0.017586 | -1.698 |
| Life stage (sub adult) | -0.08522 | 0.015736 | -5.416 |
| Life stage (adult) | -0.12769 | 0.024555 | -5.2 |
| Spring temperature | -0.11685 | 0.029431 | -3.97 |
| Spring discharge | -0.18299 | 0.057451 | -3.185 |
| Summer discharge | 0.082179 | 0.045305 | 1.814 |
| Antecedent discharge | -0.05275 | 0.020842 | -2.531 |
| Spring discharge variability | 0.09579 | 0.051971 | 1.843 |
| Summer discharge variability | 0.0862 | 0.049241 | 1.751 |
| Life stage (sub adult) : Spring temperature | 0.087758 | 0.026619 | 3.297 |
| Life stage (adult) : Spring temperature | 0.189261 | 0.028528 | 6.634 |
| Life stage (sub adult) : Spring discharge | 0.047389 | 0.050365 | 0.941 |
| Life stage (adult) : Spring discharge | 0.347838 | 0.055775 | 6.236 |
| Life stage (sub adult) : Summer discharge | -0.03305 | 0.039603 | -0.835 |
| Life stage (adult) : Summer discharge | 0.148268 | 0.046164 | 3.212 |
| Life stage (sub adult) : Antecedent discharge | 0.01642 | 0.020686 | 0.794 |
| Life stage (adult) : Antecedent discharge | 0.048111 | 0.020788 | 2.314 |
| Life stage (sub adult) : Spring discharge variability | -0.11651 | 0.053204 | -2.19 |
| Life stage (adult) : Spring discharge variability | -0.13376 | 0.053217 | -2.513 |
| Life stage (sub adult) : Summer discharge variability | -0.00538 | 0.047959 | -0.112 |
| Life stage (adult) : Summer discharge variability | -0.00568 | 0.047596 | -0.119 |
| *Random effects* |  |  |  |
| SD(Fish ID) | 0.089 |  |  |
| SD(Age\|Fish ID) | 0.193 |  |  |
| Cor(Age\|Fish ID) | 0.320 |  |  |
| SD(Capture Location) | 0.030 |  |  |
| SD(Capture Location:Year) | 0.089 |  |  |
| **Reach scale model (movement inclusive)** |  |  |  |
| *Fixed effects* |  |  |  |
| Intercept | -1.61186 | 0.024686 | -65.294 |
| Age | -0.71103 | 0.024492 | -29.031 |
| Age class | -0.03136 | 0.017116 | -1.832 |
| Life stage (sub adult) | -0.09993 | 0.01565 | -6.385 |
| Life stage (adult) | -0.14473 | 0.024328 | -5.949 |
| Spring temperature | -0.06413 | 0.02576 | -2.489 |
| Spring discharge | -0.0923 | 0.055159 | -1.673 |
| Summer discharge | 0.007803 | 0.043187 | 0.181 |
| Antecedent discharge | -0.07043 | 0.020289 | -3.471 |
| Spring discharge variability | 0.06223 | 0.045395 | 1.371 |
| Summer discharge variability | 0.053223 | 0.024729 | 2.152 |
| Life stage (sub adult) : Spring temperature | 0.12435 | 0.026894 | 4.624 |
| Life stage (adult) : Spring temperature | -0.00753 | 0.051408 | -0.147 |
| Life stage (sub adult) : Spring discharge | 0.261615 | 0.055777 | 4.69 |
| Life stage (adult) : Spring discharge | -0.02244 | 0.040295 | -0.557 |
| Life stage (sub adult) : Summer discharge | 0.150374 | 0.045041 | 3.339 |
| Life stage (adult) : Summer discharge | 0.042079 | 0.019822 | 2.123 |
| Life stage (sub adult) : Antecedent discharge | 0.072744 | 0.020564 | 3.538 |
| Life stage (adult) : Antecedent discharge | -0.03033 | 0.044956 | -0.675 |
| Life stage (sub adult) : Summer discharge variability | 0.000404 | 0.044864 | 0.009 |
| Life stage (adult) : Summer discharge variability | -1.61186 | 0.024686 | -65.294 |
| *Random effects* |  |  |  |
| SD(Fish ID) | 0.088 |  |  |
| SD(Age\|Fish ID) | 0.195 |  |  |
| Cor(Age\|Fish ID) | 0.306 |  |  |
| SD(Natal Origin) | 0.028 |  |  |
| SD(Predicted Location) | 0.000 |  |  |
| SD(Predicted Location:Year) | 0.087 |  |  |


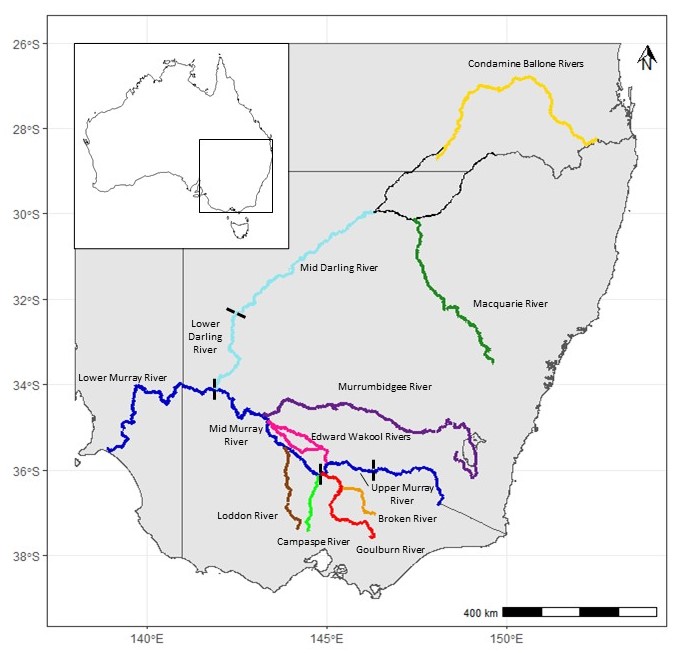
Supp. Figure 1. Map of the Murray-Darling Basin in south-eastern Australia. Colours represent river reaches identified as having different Sr compositions that fish moved among (Zampatti *et al.* 2019). Reaches with different Sr compositions within the Murray River (dark blue) and Darling River (light blue) are denoted by thick black lines. Thin black river reaches show how the Condamine-Ballone Rivers and Macquarie River are connected to the other reaches in the MDB.


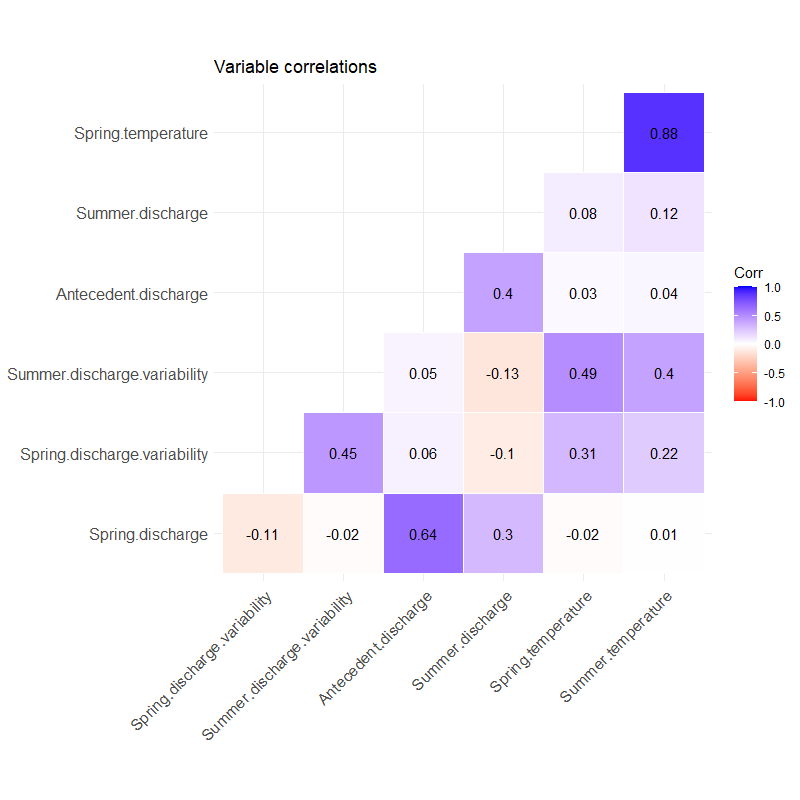


Supp. Figure 2. Correlations between environmental predictors used in analyses. The opaqueness of the squares represents higher correlation coefficients. Blue squares are positive correlations and red squares are negative correlations. Note that due to high collinearity, we did not include spring temperature and summer temperature in the same growth model.


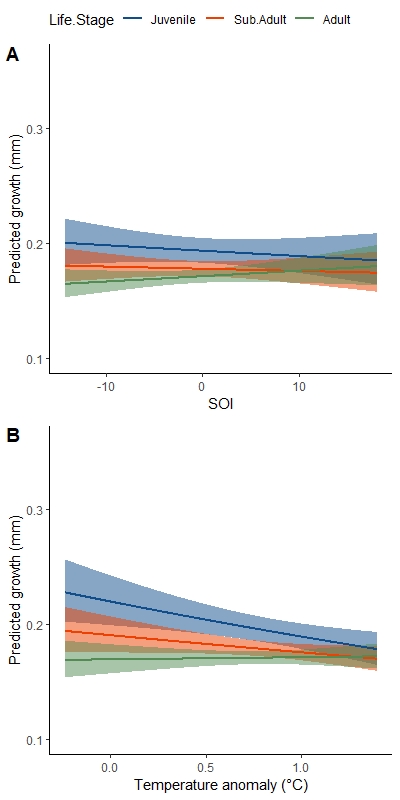


Supp. Figure 3. The predicted effect (+/- 95% CI) of (A) southern oscillation index (SOI) and (B) basin-wide temperature anomaly for the MDB on life stage-specific golden perch growth (otolith increment, mm), as derived from the best basin-scale model. The blue lines are juvenile growth, the orange lines are sub-adult growth, and the green lines are adult growth.


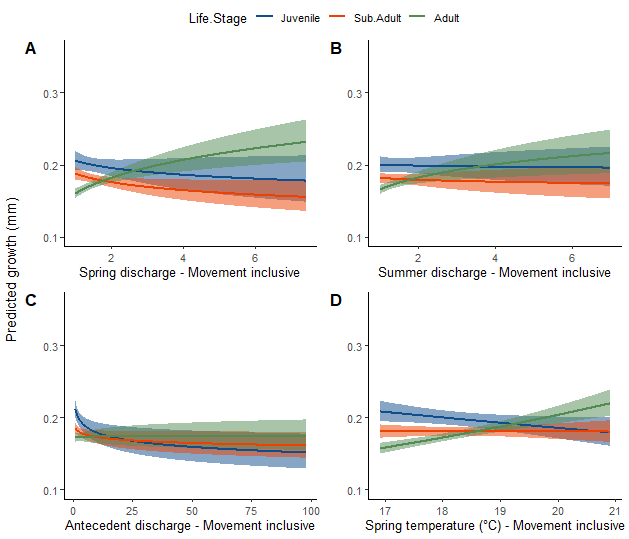


Supp. Figure 4. The predicted effect (+/- 95% CI) of (A) spring discharge, (B) summer discharge, (C) antecedent discharge, and (D) spring temperature on life stage-specific golden perch growth (otolith increment, mm), as derived from the best reach-scale (movement inclusive) model. The blue lines are juvenile growth, the orange lines are sub-adult growth, and the green lines are adult growth.


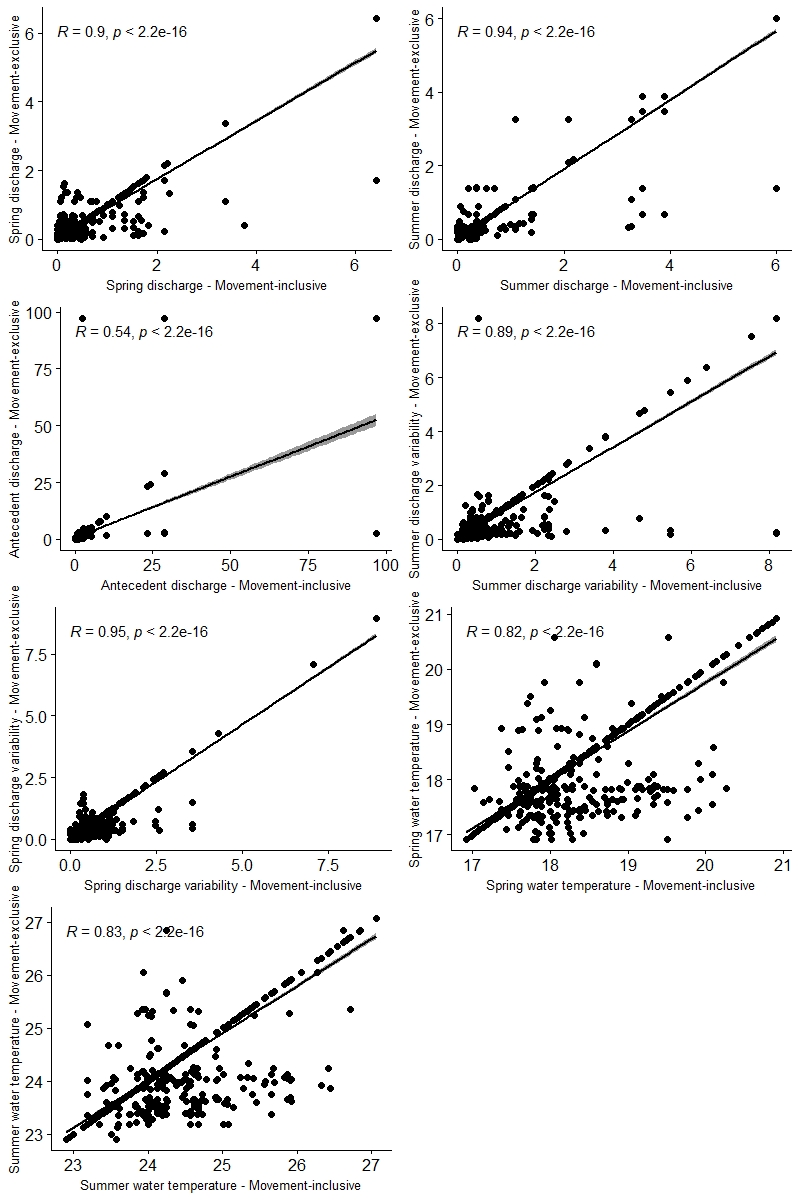


Supp. Figure 5. Correlations between the same environmental variables from the reach scale (movement exclusive) and reach scale (movement inclusive models), including Pearson’s correlation coefficient (R) and a regression line (black line). Each circle corresponds to the environmental conditions that are associated with a growth increment.


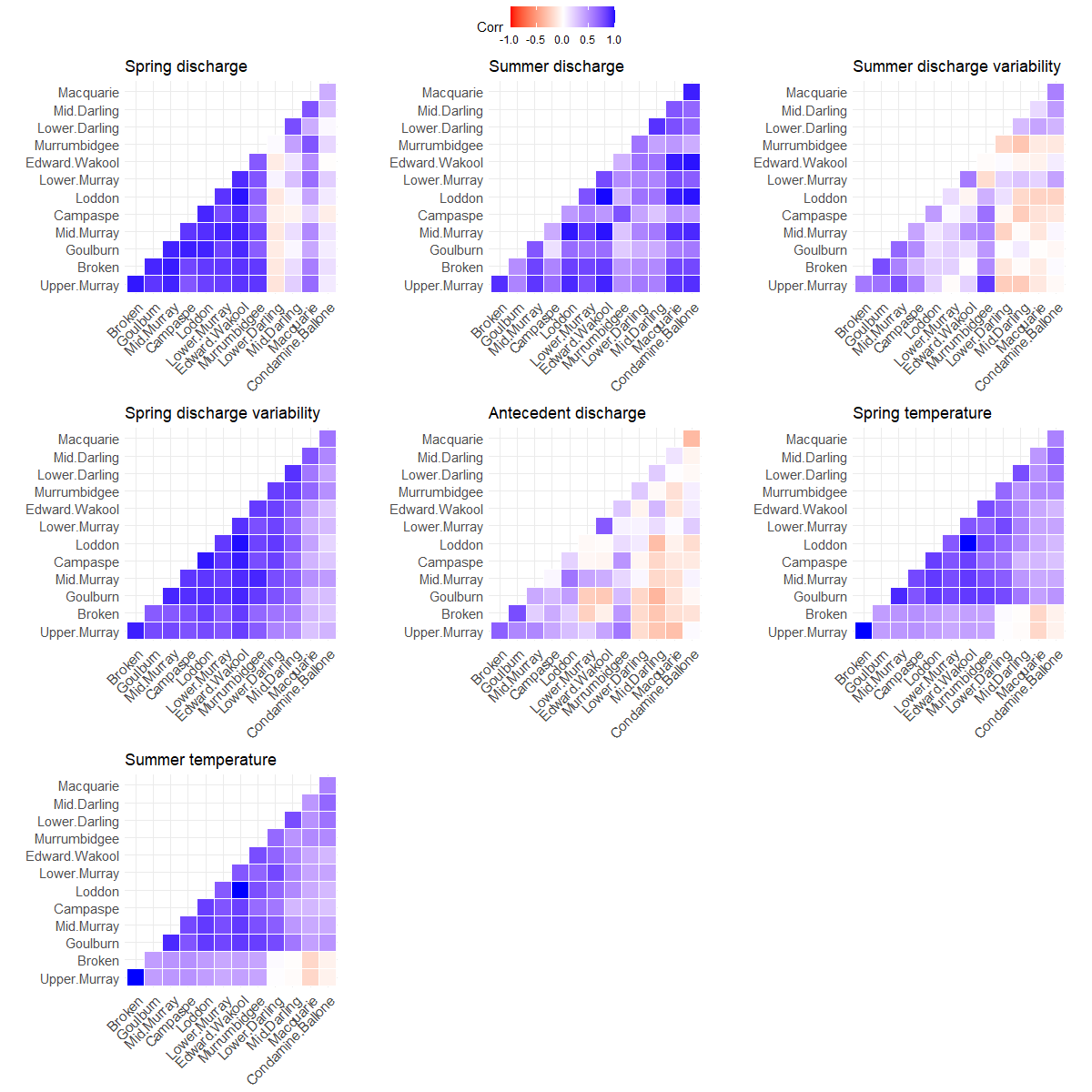
Supp. Figure 6. Spatial correlations between environmental variables across locations. Blue squares are positive correlations and red squares are negative correlations, with more opaque squares representing stronger correlation coefficients.
